# Visualizing tumor angiogenesis and boundary with polygon-scanning multiscale photoacoustic microscopy

**DOI:** 10.1016/j.pacs.2022.100342

**Published:** 2022-02-23

**Authors:** Zhiqiang Xu, Yinhao Pan, Ningbo Chen, Silue Zeng, Liangjian Liu, Rongkang Gao, Jianhui Zhang, Chihua Fang, Liang Song, Chengbo Liu

**Affiliations:** a Research Laboratory for Biomedical Optics and Molecular Imaging, CAS Key Laboratory of Health Informatics, Shenzhen Institute of Advanced Technology, Chinese Academy of Sciences, Shenzhen 518055, China; b College of Mechanical and Electrical Engineering, Guangzhou University, Guangzhou 510006, China; c Department of Hepatobiliary Surgery I, Zhujiang Hospital, Southern Medical University, Guangzhou 510280, China

**Keywords:** Multiscale photoacoustic microscopy, High speed, Polygon scanner, Distortion correction, Melanoma imaging

## Abstract

Recently, we developed an integrated optical-resolution (OR) and acoustic-resolution (AR) PAM, which has multiscale imaging capability using different resolutions. However, limited by the scanning method, a tradeoff exists between the imaging speed and field of view, which impedes its wider applications. Here, we present an improved multiscale PAM which achieves high-speed wide-field imaging based on a homemade polygon scanner. Encoder trigger mode was proposed to avoid jittering of the polygon scanner during imaging. Distortions caused by polygon scanning were analyzed theoretically and compared with traditional types of distortions in optical-scanning PAM. Then a depth correction method was proposed and verified to compensate for the distortions. System characterization of OR-PAM and AR-PAM was performed prior to *in vivo* imaging. Blood reperfusion of an *in vivo* mouse ear was imaged continuously to demonstrate the feasibility of the multiscale PAM for high-speed imaging. Results showed that the maximum B-scan rate could be 14.65 Hz in a fixed range of 10 mm. Compared with our previous multiscale system, the imaging speed of the improved system was increased by a factor of 12.35. *In vivo* imaging of a subcutaneously inoculated B-16 melanoma of a mouse was performed. Results showed that the blood vasculature around the melanoma could be resolved and the melanoma could be visualized at a depth up to 1.6 mm using the multiscale PAM.

## 1 Introduction

Photoacoustic tomography (PAT) is a powerful biomedical imaging technique based on the photoacoustic effect and has been successfully applied in many preclinical and clinical applications [1], [2], [3], [4], [5], [6], [7]. Photoacoustic microscopy (PAM), which is one of the major implementations of PAT, has achieved spatial resolutions ranging from sub-micrometers to sub-millimeters and imaging depths ranging from a few hundred micrometers to a few millimeters [8], [9], [10]. Due to its excellent scalability, PAM is widely utilized in *in vivo* imaging at multiple scales, from organelles to organs [11], [12], [13], [14], [15], [16], [17]. In PAM, both optical excitation and ultrasonic detection are focused to maximize sensitivity. Depending on whether the optical or acoustic focusing is tighter than the other one, PAM can be further classified into optical-resolution PAM (OR-PAM) and acoustic-resolution PAM (AR-PAM). OR-PAM achieves superior optical-diffraction-limited lateral resolution with a maximum imaging depth of approximately 1 mm and is typically used to image superficial vasculatures [18], [19], [20]. AR-PAM has a compromised resolution but with a much deeper penetration depth (several millimeters) and thus can be employed to image deeper biological tissues under the skin [21], [22], [23].

Many research groups have implemented multiscale PAM by integrating OR-PAM and AR-PAM in a single imaging system to obtain both superficial optical resolution images and deep acoustic resolution images [24], [25], [26], [27], [28], [29]. By sharing the same optical and acoustic components, these systems can automatically generate co-registered images, facilitate imaging operations, and reduce system costs. Jiang et al. used an electrical varifocal lens to obtain a continuously tunable lateral resolution and realized both OR and AR modes with the same imaging system [27]. Wei et al. proposed a multiscale PAM system with different spatial resolutions and maximum penetration depths based on a ring-shaped focused ultrasound transducer with two independent central frequencies [28]. Both of the aforementioned multiscale systems realized a mechanical scanning of samples through linear translation stages, which had a simple setup, precise displacement accuracy, and wide scanning range.

However, it is difficult to achieve a high scanning speed using a mechanical scanning method based on the linear translation stage. As a result, the acquisition time of these multiscale systems is too long, and none of them can be used to acquire dynamic information of *in vivo* biological tissues. Optical scanning methods based on microelectromechanical system (MEMS) scanners have proven to be very effective and have been adopted by several multiscale PAM systems to increase imaging speed [24], [29]. Mohesh et al. reported the use of a high-speed MEMS scanner for both OR-PAM and AR-PAM and developed a high-speed multiscale PAM that combines a MEMS scanner and raster mechanical movement [24]. Recently, we implemented a multiscale PAM using a fast MEMS scanner, and the imaging speed could reach 50 k A-lines per second [29]. Different with the above multiscale PAM systems which deliver the laser beam through a single-mode or multimode fiber and thus limit the amount of laser excitation energy on the sample, we adopted a free-space light transmission approach to enable higher laser transmission efficiency.

Although the imaging speed of these multiscale PAMs can be improved considerably using a MEMS scanner, a tradeoff exists between the maximum scanning frequency, mirror size, and maximum deflection angle of the MEMS scanner. If a higher frequency is required, the size of the MEMS mirror must be reduced, and thus the light delivery efficiency will be affected. For a given MEMS scanner, the scanning range is reduced with an increase in the driven frequency. Currently, the maximum effective scanning range of MEMS scanners adopted in PAM is approximately 2 mm [30], [31], [32]. Therefore, a motorized x/y-axis stage is still needed to move the sample for a scanning range of more than 2 mm, which greatly impedes the imaging speed.

As an alternative optical scanning method, polygon mirror scanning has proven to be a high-speed wide-field scanning method in PAM [33], [34]. It can achieve a fast scanning speed without compromising the mirror size or scanning range. Here, we propose a multiscale PAM based on a homemade polygon-scanning mirror that can have a large scanning range (approximately 10 mm) while maintaining a high-speed B-scan rate (14.65 Hz). In order to get accurate depth information, a depth correction method was proposed based on theoretical analysis of the distortion induced by the polygon scanner. Calibration and validation experiments of the depth correction algorithm were conducted before *in vivo* imaging. The blood reperfusion of an *in vivo* mouse ear was imaged continuously to demonstrate the feasibility of this high-speed scanning method. Results showed that the imaging speed could be increased by a factor of 12.35 compared to our previous MEMS scanning multiscale PAM [29]. Furthermore, *in vivo* imaging of a subcutaneously inoculated B-16 melanoma of a mouse was performed using the multiscale PAM to verify its capability for tumor angiogenesis study and tumor boundary visualization. The results show that the multiscale PAM has the potential for clinical application and evaluation of melanoma status.

## 2 Materials and methods

### 2.1 System setup

**Fig. 1:**
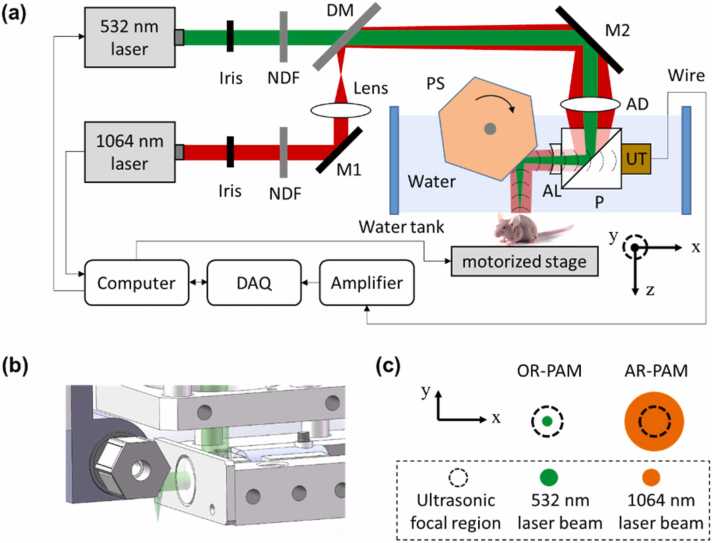
(a) Schematic of the multiscale PAM. NDF, neutral density filter; DM, dichroic mirror; AD, achromatic doublet; P, prism; AL, acoustic lens; UT, ultrasound transducer; PS, polygon-scanning mirror. (b) Structure of the polygon mirror. (c) OR and AR modes.

As Fig. 1(a) shows, the multiscale PAM system utilizes two nanosecond-pulsed lasers: a 532 nm laser (GKNQL-532–6–10, Guoke Laser Technology) and a 1064 nm laser (Pilot-30, ZY Laser Science and Technology). For each laser, the output beam is reduced to an appropriate diameter by an iris (SM1D12D, Thorlabs) and then attenuated by a neutral-density filter (NDC-50C-2M, Thorlabs). The 1064 nm beam is defocused by a plano-convex lens (f = 150 mm) and then transmitted to a dichroic mirror (DM). The two beams are combined at the DM, which transmits a 532 nm beam and reflects a 1064 nm beam. The combined beams are further deflected by a mirror (M2) to the imaging head.

In the imaging head, both the 532 nm and 1064 nm beams pass through an achromatic doublet lens (f = 50 mm) and then are reflected off by an optical/acoustic beam combiner which is made by gluing an aluminum-coated prism to an uncoated prism (#32-331 and #32-330, Edmund Optics Inc.). The aluminum coating reflects the optical beam and transmits ultrasound. The optical beam is further transmitted through an acoustic lens (#48-267-INK, Edmund Optics Inc.) and is then deflected by a polygon-scanning mirror to the sample. Finally, the generated acoustic beam is collimated by the acoustic lens, transmitted through the optical/acoustic combiner, and detected by a broadband piezoelectric transducer (50-MHz center frequency, 70% bandwidth, V214-BB-RM, Olympus). The optical/acoustic combiner, transducer, and bottom half of the polygon mirror are immersed in water in a homemade water tank. A thin layer of ultrasound gel is applied between the object to be imaged and the transparent polyethylene membrane at the bottom of the water tank for acoustic coupling.

As Fig. 1(b) shows, the polygon-scanning mirror has six aluminum-coated surfaces that reflect both light and acoustic beams. Each surface has a width of 5 mm and a length of 8 mm. The circumcircle diameter of the polygon is 14 mm. A water-immersible brushless DC-motor is axially assembled with the center aperture of the polygon mirror to drive the mirror. The polygon mirror is immersed in water to maintain the confocal scanning of optical and acoustic beams for high detection sensitivity, and six repeated cross-sectional scans (B-scans) can be obtained in one rotation of the motor. Volumetric imaging is achieved through hybrid scanning: fast optical scanning along the x axis using a polygon mirror and slow mechanical scanning along the y axis through a motorized linear stage. The received photoacoustic (PA) signal is amplified by 49 dB and sampled by a high-speed DAQ card (ATS9371, Alarzar Tech). The computer synchronizes the polygon mirror, motorized stage, and data acquisition card.

By setting different optical focusing in the imaging head, the 532 nm beam has a much tighter optical focus than the 1064 nm beam on the sample surface, as illustrated in Fig. 1(c). The optical focus of the 532 nm laser beam is adjusted for confocal alignment with the acoustic focus. On the sample surface, the 532 nm focused beam is much smaller than that of the acoustic focus and thus forms an OR-PAM mode. The 1064 nm beam is larger than the acoustic focus, thus forming the AR-PAM mode.

### 2.2 Scanning range test

**Fig. 2:**
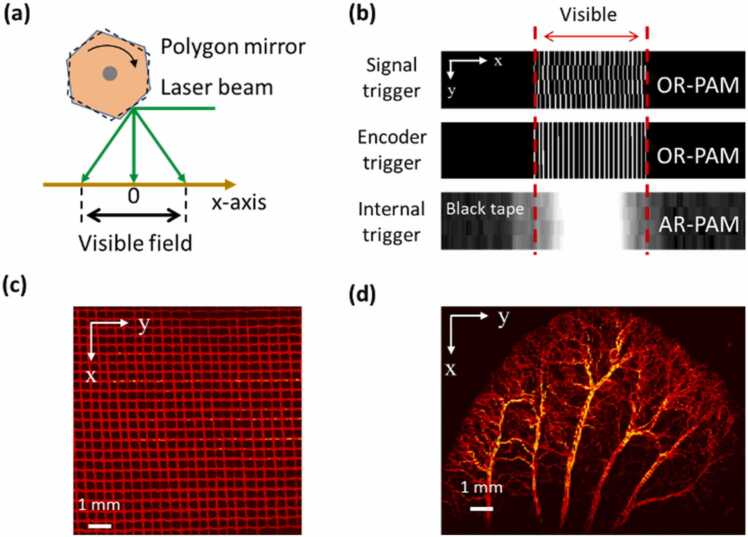
(a) Schematic of the B-scan range. (b) Original MAP images of four continuous B-scans containing both visible and invisible areas. For OR-PAM, a stainless grid with a known size was imaged at 532 nm, and the visible range was measured to be ~10 mm. The image quality could be improved by using the encoder trigger mode instead of the signal trigger mode. For AR-PAM, a black tape was imaged at 1064 nm. The measured visible range was 8.2 mm and a 10 mm area was retained to maintain the same size as in the OR-PAM results. (c) and (d) MAP images of the stainless grid sample (10 mm × 10 mm) and an *in vivo* mouse ear (10 mm × 12 mm) with OR-PAM at 532 nm.

As Fig. 1(b) shows, the polygon mirror rotates clockwise, and the laser beam will be blocked by the mechanical structure when the edge area of each B-scan is scanned. In addition, the signal-to-noise ratio (SNR) of the PA signal is reduced at both sides of each B-scan because the sample is out of focus. Therefore, only the center part of each B-scan is visible, as shown in Fig. 2(a). To quantify the visible range of B-scans in both OR-PAM and AR-PAM, phantom and *in vivo* imaging experiments were conducted.

For the OR-PAM, a stainless grid was imaged to measure the scanning range. The diameter of the stainless wire in the grid was 110 µm, and the average space between the two adjacent wires was 310 µm. A signal generator was initially used to trigger the 532 nm laser (defined as the signal trigger mode) at a frequency of 20 kHz with a B-scan rate of ~7.32 Hz. Continuous B-scans were obtained without moving the y stage, and then the top-view MAP (maximum amplitude projection) image was calculated. The result (first row in Fig. 2(b)) showed that the image quality was greatly affected by the stability of the rotation speed of the DC motor driving the polygon mirror. As the laser beam was triggered at equal time intervals by the signal generator, there was a mismatch between two adjacent B-scans when a disturbance occurred in the scanning speed.

To solve this problem, we applied a motor encoder to trigger the laser source (defined as the encoder trigger mode). The encoder of the motor that drives the polygon-scanning mirror outputs 16384 pulses sequentially in one circle, which corresponds to a table of evenly distributed rotation angles one by one. When the encoder trigger mode was applied, each PA signal was generated at a specific position along the x axis when the polygon mirror was rotated by 0.022°. Therefore, the problem derived from unstable rotation speed could be avoided. The results (second row in Fig. 2(b)) showed that jitter occurring in the signal trigger mode could be avoided through the encoder trigger mode, and the reconstructed image agreed with the grid sample. In the MAP image, 24 stainless wires could be resolved clearly, from which the visible range of OR-PAM was measured to be 10.1 mm and the average step size was 8.2 µm. The encoder trigger mode was used and the visible scanning range was set to 10 mm for OR-PAM in the following experiments.

For AR-PAM, because the SNR of the PA signal generated by the stainless grid was poor, a black tape was imaged instead to evaluate the scanning range. Note that the 1064 nm laser source can be triggered only by its controller (defined as the internal trigger mode) and does not support external trigger modes such as the signal or encoder triggers previously mentioned. Because of the different trigger modes allowed by the lasers (encoder trigger mode for 532 nm laser, internal trigger mode for 1064 nm laser), the multiscale imaging of biological tissues was realized by performing OR-PAM imaging first and then AR-PAM with the same imaging system. In the imaging test of AR-PAM, the B-scan rate was set to 7.32 Hz and the pulse repetition rate (PRR) was set to 3.7 kHz. Continuous B-scans were obtained without moving the y stage, and then the top-view MAP images were calculated from the acquired data. The results (bottom row in Fig. 2(b)) showed that the pixel jitter was negligible as compared with the lower resolution of AR-PAM. The measured visible range was 8.2 mm and the average step size was 42 µm. For AR-PAM, the laser beam size will increase significantly when the scanning angle is very large which will lead to a reduced laser fluence and a lower SNR of PA signal. Therefore, the visible range of AR-PAM is small than that of OR-PAM. To maintain the same size as OR-PAM to generate co-registered images, the visible scanning range for AR-PAM was set to 10 mm in the following experiments.

Both phantom and *in vivo* imaging experiments were conducted at 532 nm to further demonstrate the scanning range of the system. All animal experimental procedures as described in this section and those that follow were approved by the Institutional Animal Care and Use Committee of Shenzhen Institute of Advanced Technology, Chinese Academy of Sciences. Fig. 2(c) shows the reconstructed image of the stainless grid (10 mm × 10 mm), which was generally uniform, and the PA signal amplitude was approximately consistent over a wide scanning range. Fig. 2(d) shows the results of *in vivo* imaging of a mouse ear (10 mm × 12 mm, BALB/c, eight weeks old, male), which indicates the scanning range was well suited for the large size of an entire mouse ear.

### 2.3 Depth correction

The optical scanner helps enhance the imaging speed of PAM, however, they may also introduce image distortions [35], [36], [37], [38]. In round-trip optical-scanning PAM using MEMS scanner or galvanometer, there are two main distortions:

(1) A non-uniform distortion of top-view MAP images caused by the non-uniform scanning speed [38]. During the round-trip scanning of a MEMS scanner or galvanometer, the scanning speed reaches the highest in the middle and decreases to zero at both distal ends. The laser source is triggered at an equal time interval. Thus the data sampling is inevitably non-uniform in the imaging region. In addition, the scanning mirror is often driven by a sinusoidal signal to achieve rapid scanning, which will further aggravate the non-uniform distortion. This distortion can be corrected by several resampling algorithms during data processing [37], [38]. Likewise, it can also be avoided by triggering the laser source using the encoder trigger mode proposed in this paper.
(2) A bending distortion of B-scan images caused by curved scanning due to deflection of laser beam along with different scanning angles [39]. In a post-object scanning PAM system, an optical scanner is placed after an objective lens and the focus plane of the incident laser beam is not flat because of the angled deflection of the laser beam during scanning. Therefore, the generated B-scan images will be curved if it is reconstructed in the Cartesian coordinate directly. This distortion could be avoided by using a pre-objective scanning method in which the scanner is placed before an f-theta scan lens. It could also be corrected by post-processing algorithm by converting the acquired data from polar coordinate to Cartesian coordinate [39], [40].

**Fig. 3:**
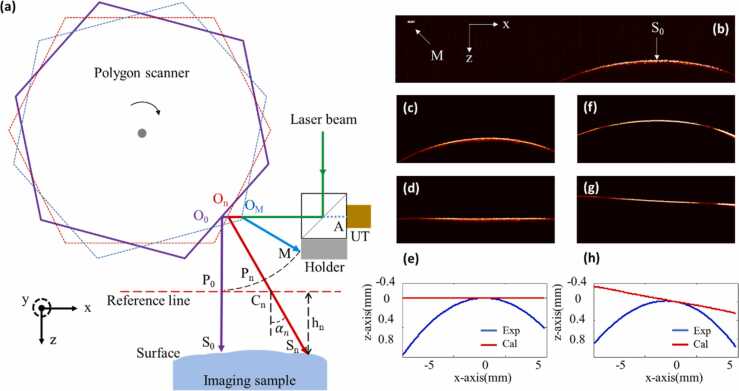
(a) Illustration of the depth distortion caused by polygon scanner during imaging; UT, ultrasound transducer. (b) A whole B-scan image (containing both visible and invisible area) of a black tape attached to a cover glass. (c) B-scan image (only the visible area is reserved) of the black tape placed horizontally under the imaging probe. (d) Depth calibration of (c). (e) Contour line of the black tape extracted from (c) and (d) respectively. (f) B-scan image of the black tape placed obliquely under the imaging probe. (g) Depth calibration of (f). (h) Contour line of the black tape extracted from (f) and (g) respectively. All the data were obtained with OR-PAM at 532 nm.

As an optical scanning method, the polygon scanner in the multiscale PAM system will also introduce distortions but with specific features:

(1) The non-uniform distortion mentioned above is not obvious in the polygon-scanning system. Different with round-trip scanners, the polygon mirror rotates in one direction with a relatively constant speed. Even though there is random disturbance of the rotation speed which may cause jittering of the scanner, it causes far less interference compared to the non-uniform scanning speed in round-trip scanners. Furthermore, the proposed encoder trigger method in this study can effectively avoid the jittering and ensure a uniform space sampling. Therefore, the non-uniform distortion of top-view MAP images is not obvious in our system, which could be confirmed by Fig. 2(c) and (d).
(2) As shown in Fig. 3(b), polygon scanning leads to serious bending distortion in the B-scan image. This bending distortion is different from the one caused by round-trip scanners. In round-trip optical-scanning PAMs, the laser beam deflects at a fixed position (*i.e*., center of the scanning mirror). For such cases, a standard polar coordinate can be easily established based on the deflection angle and the transmission time of PA signal. Then the bending distortion can be corrected with coordinate transformation. However, in the polygon-scanning PAM, the polygon scanner rotates around the center of the hexagon instead of the center of each mirror, meaning that the reflection position cannot remain fixed. The polar coordinate in round-trip scanning is not suitable for polygon scanning, and a dedicated correction method is needed.

We developed such a correction method based on theoretical analysis first. As shown in Fig. 3(a), in the X-Z plane, the polygon scanner rotates clockwise and the laser is triggered at each rotation angle. To distinguish PA signals generated at different angles, the polygon scanner and the laser beam are marked with different colors, as illustrated in Fig. 3(a). The point where the laser beam is incident on the surface of the sample is defined as $S_{n}(n=0,1,\ldots ,1365)$, among which $S_{0}$ represent the position where the laser beam is incident perpendicularly. The angle between the laser beam and Z-axis is defined as$\;\alpha_{n}$, which is a set of known constants. The reflection point of an incident laser beam on the polygon scanner is defined as$\;O_{n}$. The generated PA signal will propagate in the opposite direction of the incident light, being reflected by the polygon scanner and finally detected by the UT. The center point on the detection surface of the UT is marked as point A. The length of transmission path for PA signal is defined as $d_{n}$, which could be calculated from the acquired A-line data. From Fig. 3(a), we can obtain:

(1) $$d_{n}=S_{n}O_{n}+\;O_{n}A$$

where $S_{n}O_{n}$ denotes the length between the two points ($S_{n}{\mathrm{and O}}_{n}$). The denotation of a length using two points is applied in the following Equations. A metal holder adhered to the optical/acoustic beam combiner is used to generate reference PA signals during imaging. The point where the laser beam is incident on the surface of the holder is marked as point M. Because the metal holder is fixed on the imaging probe, the origin of the reference PA signal is known in each raw B-scan image, as shown in Fig. 3(b). The sequential order of A-lines during imaging is also known thus the reference PA signal of the metal holder can be utilized to locate PA signal generated at $S_{0}$ in each raw B-scan data. The length of the transmission path for the metal holder’s PA signal is defined as L, which is a constant and can be obtained:

(2) $$L=MO_{m}+\;O_{m}A$$

$P_{n}$ is a point selected on line $S_{n}O_{n}$ which satisfies the formula:

(3) $${L=P}_{n}O_{n}+\;O_{n}A$$

The Z-axis coordinate of $P_{0}$ is set as “0” and X-axis across $P_{0}$ is defined as a reference line (red dashed line in Fig. 3(a)). The intersection of this reference line and $S_{n}O_{n}$ is defined as point $C_{n}$. The distance between $P_{n}$ and $C_{n}$ is defined as $m_{n}$:

(4) $$m_{n}=C_{n}P_{n}$$

The depth value (*i.e*., Z-axis coordinate) of point $S_{n}$ is defined as $h_{n}$ which is the distance from $S_{n}$ to the reference line. A negative or positive value denotes that the point is above or below the reference line. From Fig. 3(a), we can obtain:

(5) $$h_{n}=S_{n}C_{n}*\cos\left(\alpha_{n}\right)$$

(6) $$S_{n}C_{n}=\;S_{n}O_{n}+\;O_{n}A-\left(P_{n}O_{n}+\;O_{n}A\right)-C_{n}P_{n}$$

By combing Eqs. (1), (3), (5) and (6), the depth value of point $S_{n}\;$is derived in the following formula:

(7) $$h_{n}=(d_{n}-L-m_{n})*\cos\left(\alpha_{n}\right)$$

where L and $\alpha_{n}$ are known constants; $d_{n}$ can be calculated from acquired raw data; $m_{n}$ is unknown which needs to be measured before the calculation of $h_{n}$.

For the determination of$\;m_{n}$, we conducted a calibration test first. A black tape attached on the top surface of a cover glass was imaged. The black tape was placed horizontally beneath the imaging probe and a whole B-scan image containing both visible and invisible area was obtained with OR-PAM, as shown in Fig. 3(b). The depth (*i.e*., z-axis coordinate) of each A-line was detected and then a contour line of the B-scan was extracted by curve fitting. Because the black tape was placed horizontally, the depths of PA signals ($h_{0},h_{1},\cdots {,h}_{n}$) for each point ($S_{0},S_{1},\cdots {,S}_{n}$) at the target surface are equal, thus:

(8) $$h_{n}=h_{0}$$

By combing Eqs. (7) and (8), $m_{n}$ can be calculated:

(9) $$m_{n}=d_{n}-L-\frac{d_{0}-L}{\cos\left(\alpha_{n}\right)}$$

Then the depth of each PA signal $h_{n}$ could be calculated by Eq. (7). The depth correction of B-scan image was completed by shifting each A-line data according to the depth of PA signal in the Z-axis. As shown in Fig. 3(c), (d) and (e), the contour line of the black tape after depth correction is flat and horizontal, which is authentic to its true state.

A verified experiment was then conducted to demonstrate the feasibility of the depth correction method. In the experiment, the black tape mentioned above was placed tilted under the imaging probe. As shown in Fig. 3(f), the raw B-scan image is curved. After depth correction, the B-scan image becomes flat and the contour line is an oblique straight line which is consistent with the real condition.

The calibration and validation experiments were the same for the depth correction of AR-PAM. All the B-scan images in the following experiments were obtained by applying the depth correction method.

### 2.4 Spatial resolution and maximum imaging depth test

**Fig. 4:**
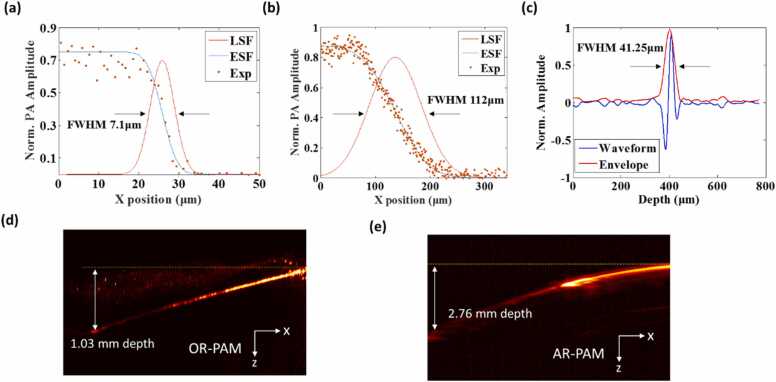
(a) Lateral resolution of the OR-PAM. (b) Lateral resolution of the AR-PAM. (c) Axial resolution of the multiscale PAM. (d) and (e) Maximum imaging depths of OR-PAM and AR-PAM, respectively.

The theoretical lateral resolutions of OR-PAM and AR-PAM were calculated to be 4.5 and 72.9 µm, respectively. The actual lateral resolution was experimentally quantified by scanning the sharp edge of a blade placed at the center of the B-scan. The edge spread function was then acquired and fitted to derive the line spread function (LSF). The full width at half maximum (FWHM) of the LSF was measured as the lateral resolution. As Fig. 4(a) and (b) show, the lateral resolution of OR-PAM and AR-PAM were estimated to be 7.1 and 112 µm, respectively. The degradation of the experimentally measured lateral resolutions as compared to the theoretical values was presumably caused by the optical aberration induced by the optical elements and the defocusing at oblique angles induced by the polygon-scanning mirror.

For axial resolution, both OR-PAM and AR-PAM share the same value, which depends on the detection bandwidth of the ultrasonic transducer. A 10-μm wide tungsten wire, the diameter of which was much smaller than the acoustic wavelength, was imaged with OR-PAM at 532 nm to measure the axial resolution of the imaging system. The FWHM of the Hilbert-transformed envelope of the PA signal was measured to be 41.25 µm (Fig. 4(c)), which is close to the theoretical value of 38.7 µm.

To measure the maximum imaging depth of the imaging system, a tungsten wire and plastic tube filled with a nanoparticle probe (absorption peak is 1064 nm) were inserted obliquely into a fresh chicken breast and then imaged. The tungsten wire was imaged with OR-PAM, and the measured maximum depth was 1.03 mm (Fig. 4(d)). The 1064 nm nanoparticle probe was imaged with AR-PAM and the maximum imaging depth was 2.76 mm (Fig. 4(e)).

## 3 Results and discussion

### 3.1 Blood reperfusion experiment

**Fig. 5:**
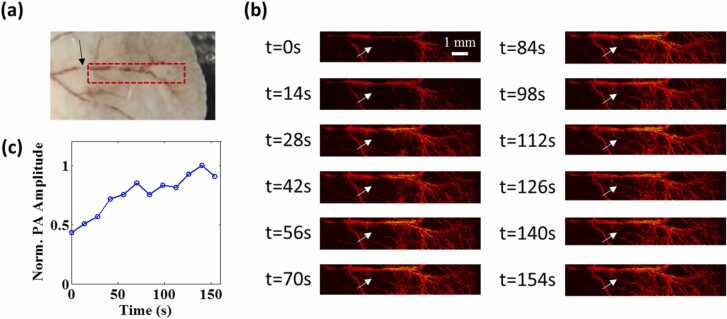
High-speed imaging of blood reperfusion after vascular compression over a 10 mm × 2 mm area in a mouse ear using OR-PAM. (a) Photograph of the imaging area. The black arrow points to the vascular compression position using a cotton swab prior to imaging. (b) Changes in MAP images at different times. The baseline is the image at time 0 when the compression cotton swab was removed. The white arrow indicates the vascular reperfusion in 3 min (c) Changes in PA amplitude at the position marked by the white arrow in (b).

To demonstrate the fast imaging capacity based on the polygon-scanning mirror, a continuous imaging experiment was designed to observe the dynamic change in blood perfusion in a nude mouse ear (BALB/c, eight weeks old, male). Throughout the imaging experiment, the mouse was kept under anesthesia with 1% isoflurane. The mouse was placed on an experimental stage, and the body temperature was maintained at 37 °C using a heating pad. Prior to imaging, the root area of the artery vessels (black arrow in Fig. 5(a)) was compressed using a cotton swab to block blood flow for 30 s. The ear was imaged immediately after the cotton swab was withdrawn. The blood reperfusion process could be observed by OR-PAM at 532 nm over a 10 mm × 2 mm region (see Visualization 1). The PRR of the laser was set as 40 kHz, and the B-scan rate was 14.65 Hz. It took 14 s to acquire each frame in the continuous imaging. As Fig. 5(b) shows, the baseline was the image at time 0, and the vessel marked by the white arrow was invisible because of ischemia. The recovery of blood perfusion started from the two sides of the vessel. Quantitative analysis of the PA amplitude (Fig. 5(c)) showed that the recovery of blood perfusion occurred very quickly in the beginning, and the PA amplitude gradually returned to normal within 3 min

Supplementary material related to this article can be found online at doi:10.1016/j.pacs.2022.100342.

The following is the Supplementary material related to this article Visualization 1..

Visualization 1

### 3.2 Melanoma imaging experiment

**Fig. 6:**
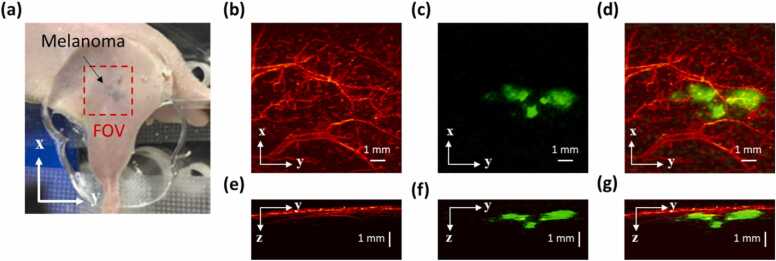
*In vivo* OR and AR images of a subcutaneously inoculated B16-melanoma in a nude mouse using multiscale PAM. (a) Photograph of the melanoma. (b) and (e) Top and side view MAP images, respectively, obtained by OR-PAM at 532 nm. (c) and (f) Top and side view MAP images, respectively, obtained by AR-PAM at 1064 nm. (d) and (g) Merged images of the top and side view MAP images, respectively, with OR-PAM and AR-PAM.

Angiogenesis is known to be one of the major symptoms during tumor growth and its imaging has been demonstrated to be useful for tumor diagnosing and therapy assessment [41], [42]. Multiscale PAM is very suitable for *in vivo* imaging of the skin tumor: tumor boundary can be visualized by AR-PAM, which has a deeper penetration; tumor angiogenesis can be resolved by OR-PAM, which has a higher resolution. Finally, the morphological relationship between the tumor and surrounding vasculature can be revealed by combining the aforementioned two co-registered images. As a proof of concept, a subcutaneously inoculated B16-melanoma in an immunocompromised nude mouse was imaged. Prior to imaging, melanoma B16 cells were subcutaneously injected into a nude mouse (BALB/c, eight weeks old, male) on the body side near the hind leg to grow in one week. A 10 mm × 10 mm region around the melanoma was imaged. The results are shown in Fig. 6, in which the blood vessels are pseudo-colored red and the melanoma is pseudo-colored green. For OR-PAM, the vascular-containing microvessels on the surface of the tumor could be imaged at 532 nm with high resolution (Fig. 6(b)), whereas the imaging depth was limited (Fig. 6(e)). For AR-PAM, because the melanoma had a much higher absorption than blood and water at 1064 nm, the top-view MAP of the melanoma could be acquired with good contrast (Fig. 6(c)). The maximum imaging depth of AR-PAM was much higher because of the weak beam focusing and higher laser pulse energy, which enabled the boundaries of the melanoma to be visualized. Fig. 6(f) shows that both the top and bottom portions of the melanoma with 1.6 mm thickness could be imaged under the skin. The merged top and side MAP images of OR-PAM and AR-PAM are shown in Fig. 6(d) and (g), respectively, from which the shape and location of the melanoma can be clearly observed.

Compared with our previous multiscale PAM, this new system can shorten the imaging time from 14 min to 68 s when imaging a 10 mm × 10 mm area with the same step size. Hence the imaging time is shortened by a factor of 12.35. In our previous multiscale PAM, the scanning range in B-scan direction is quite limited (1 mm). When imaging a 10 mm × 10 mm area, multiple sub-regional images were obtained first and then stitched together in the B-scan direction. However, our new system has a B-scan range of 10 mm, and therefore no stitching is needed in B-scan direction. We used a motorized stage in our previous system to move the imaging head to achieve stitched imaging. The movement of the motorized stage is slow and therefore takes up majority of the imaging time. In our new system, without the stitching process, the movement time is saved. This rapid imaging capability facilitates its potential application in the clinical detection and evaluation of melanoma.

## 4 Conclusion

In this study, we developed a multiscale PAM that can visualize tumor boundaries at high speed using a polygon-scanning mirror. The imaging speed of the multiscale PAM was improved by a factor of 12.35 as compared with our previous multiscale PAM using a MEMS scanning mirror. A depth correction method was proposed based on theoretical analysis of the distortion induced by the polygon scanner. The accuracy of the correction method was verified through phantom experiment. A continuous imaging experiment was designed to observe the dynamic change in blood perfusion in a nude mouse ear to demonstrate the fast imaging capacity. An *in vivo* melanoma imaging experiment showed that the blood vasculature around the melanoma at the surface could be resolved and the melanoma boundaries could be detected at a depth up to 1.6 mm. Multiscale PAM was determined to be suitable for *in vivo* tumor status visualization, and its rapid imaging capability facilitates its future clinical applications.

The imaging speed of the multiscale system can be further increased by using a laser with higher pulse repetition rate (*e.g.*, 1 MHz). Due to the limited pulse repetition rate of the current laser source, the rotation speed of the motor that drives the polygon mirror was set at 146 r/min and the B-scan rate was 14.65 Hz in the experiment. When much faster pulsed laser is used, the rotation speed of the motor can be set at the recommended value (5980 r/min) and a much higher B-scan speed can be achieved (~600 Hz). The imaging speed therefore can be improved by 500 times compared with our previous multiscale system based on MEMS mirror. Besides that, the image quality could also be improved because of the better stability of the polygon scanning at the recommended rotation speed of the motor. In addition, there exists manufacturing error for the homemade polygon scanner which causes inconsistencies in the six B-scans in one rotation. Therefore, the image quality will be further improved by adopting polygon mirrors with higher machining accuracy. Functional imaging with a multi-wavelength laser such as a Raman laser source will enhance the capability of multiscale PAM for the visualization of tumor functions for which hypermetabolism and hypoxia are quintessential hallmarks.

## Declaration of Competing Interest

The authors declare that there are no conflicts of interest.
